# Repeat and Relapsing Peritonitis Microbiological Trends and Outcomes: A 21-Year Single-Center Experience

**DOI:** 10.1155/2021/6662488

**Published:** 2021-01-30

**Authors:** Marina Reis, Catarina Ribeiro, Ana Marta Gomes, Clara Santos, Daniela Lopes, João Carlos Fernandes

**Affiliations:** Nephrology Department, Centro Hospitalar Vila Nova de Gaia/Espinho, Vila Nova de Gaia, Portugal

## Abstract

Peritonitis is a major peritoneal dialysis complication. Despite a high cure rate, relapsing and repeat peritonitis is associated with Tenckhoff catheter biofilm and multiple episodes of peritoneal damage. In relapsing peritonitis, prompt catheter removal is mandatory; otherwise, in repeat peritonitis, there is not a clear indication for catheter removal. It is questionable if the approach to removal should be different. There are few recent data on repeat and relapsing peritonitis microbiology and clinical outcomes since most studies are from the past decade. This study evaluates the microbiology, clinical outcomes, and impact of relapsing and repeat peritonitis on technique survival and the impact of catheter removal in development of further peritonitis episodes by the same microorganism. We developed a single-center retrospective study from 1998 to 2019 that compared repeat and relapsing peritonitis with a control group in terms of causative microorganisms, cure rate, catheter removal, and permanent and temporary transfer to hemodialysis. We also compared repeat and relapsing peritonitis clinical outcomes when Tenckhoff catheter was not removed. Comparing to the control group, the repeat/relapsing group had a higher cure rate (80.4% *versus* 74.5%, *p*=0.01) and lower rate of hospitalization (10.9% versus 27.7%, *p*=0.01). Technique survival was superior in the repeat/relapsing group (log rank = 4.5, *p*=0.03). Gram-positive peritonitis was more common in the repeat/relapsing group especially *Streptococci viridans* (43.5% versus 21.3%, *p*=0.01) and Gram-negatives in the control group (26.6% vs 9.0%, *p*=0.02). When the Tenckhoff catheter was not removed after a repeat episode, 58.6% developed a new repeat/relapsing episode versus 60.0% in the relapsing group. Although repeat and relapsing peritonitis have a higher cure rate, it leads to further episodes of peritonitis and consequent morbidity. When Tenckhoff catheter was not removed, the probability of another peritonitis episode by the same microorganism is similar in repeat and relapsing peritonitis.

## 1. Introduction

Peritoneal dialysis- (PD-) related peritonitis is a major PD complication and an important cause of ultrafiltration failure and hemodialysis transfer [1, 2]. Although repeat and relapsing episodes seem to have a high cure rate, they deserve special attention because there is a substantial risk of developing further episodes, which may perpetuate peritoneal membrane damage [3, 4]. There is little evidence that supports a different approach to relapsing and repeat peritonitis once both are related to colonization of peritoneal catheter by a microorganism and are associated with multiple episodes of peritonitis [4–7]. There are few recent data on repeat and relapsing peritonitis microbiology and clinical outcomes since most studies are from the past decade [4, 8]. During the last years, there was a change on technique and microbiological policies, namely, exit-site antibiotic prophylaxis, so it is important to evaluate if the microorganisms and clinical outcomes of repeat and relapsing episodes remain the same.

This study evaluates the microbiology, clinical outcomes, and impact of relapsing and repeat peritonitis on technique survival and the impact of catheter removal in development of further peritonitis episodes by the same microorganism.

## 2. Materials and Methods

### 2.1. Patient Selection

All episodes of PD peritonitis in our unit from 1998 to 2019 were reviewed. The data included demographic information, primary kidney disease cause, comorbid conditions, PD modality, microbiology of peritonitis episodes, and cause of PD withdraw. The diagnosis of peritonitis was based on International Society of Peritoneal Dialysis (ISPD) recommendations and at least two of the following: abdominal pain and/or cloudy dialysis effluent; dialysis effluent white cell count >100/*μ*L (after a dwell time of at least 2 hours) with >50% polymorphonuclear cells; and positive dialysis effluent culture. Exit-site infection was diagnosed when there was purulent drainage from the exit site with or without erythema.

**Figure 1:**
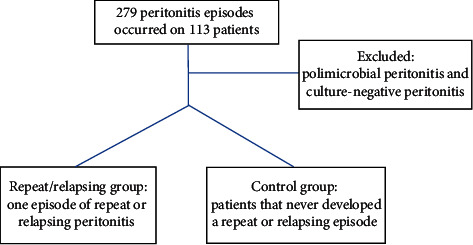
Study design.

According to ISPD guidelines, repeat peritonitis was defined as an episode that occurs more than four weeks after completion of therapy of a prior episode with the same organism and relapsing peritonitis as an episode that occurs within four weeks after completion of therapy of a prior episode with the same organism or one sterile episode.  Figure 1 shows the study design. In 21 years of the study period, 279 episodes of peritonitis were recorded: 46 episodes (35 repeat peritonitis and 11 relapsing episodes) occurred in 36 patients. The control group included 77 patients that never had a repeat or relapsing peritonitis and in these patients 94 episodes of peritonitis occurred. Polymicrobial and culture negative episodes were excluded. To compare technique survival, all patients that left the program because of transplantation or personal option were excluded.

### 2.2. Clinical Management

In our program, antibiotics are not routinely included in daily exit-site care. Since 2013, *Staphylococcus aureus* carriers (a positive nasal, exit-site, or groin swab) are submitted to a decontamination protocol with mupirocin. Carriage status is evaluated before PD starts, then every three months in patients with a history of a positive *Staphylococcus aureus* swab, and annually in patients that were never carriers. Peritonitis episodes were treated with the standard antibiotic protocol of our center at that time: cefazolin and ceftazidime from January 1998 to December 2012 and vancomycin and ceftazidime from 2013 to present.

Cure was defined as resolution of abdominal pain, clearing of dialysate, and PD effluent neutrophil count less than 100/ml. Tenckhoff catheters were removed in refractory, relapsing, fungal, refractory exit-sites, and tunnel infection peritonitis; temporary hemodialysis was performed when necessary. Simultaneous catheter removal and reinsertion has been performed after a repeat peritonitis episode since 2004. Patients were only switched to long-term hemodialysis when attempts of Tenckhoff catheter reinsertion failed because of patient fatigue/burnout or when there was ultrafiltration failure.

### 2.3. Statistical Analyses

Categorical variables were presented as frequencies and percentages and were compared with the use of Fisher's exact test or the chi-square test, as appropriate. Continuous variables are presented as means and standard deviations or medians and 25^th^ and 75^th^ percentile for variables with skewed distributions. Normal distribution was checked using the Shapiro–Wilk test as well as analysis of skewness and kurtosis. Continuous normally distributed data variables were compared with Student's *t*-test. Continuous nonnormally distributed data were compared with Mann–Whitney test. Kaplan–Meier estimates were used to construct the technique survival curves. All reported *p* values are two-tailed, with a *p* value less than 0.05 indicating statistical significance. Statistical analyses were performed by SPSS for Windows software version 23.0 (SPSS Inc., Chicago, IL).

## 3. Results

### 3.1. Population Characteristics

From 1998 to 2019, there were 279 episodes of PD-related peritonitis in 113 patients. The overall peritonitis rate was 0.49 episodes per patient-year.

We analyzed 36 patients (32.4%) with repeat and/or relapsing peritonitis: 20 patients (55.6%) had repeat peritonitis and 16 (44.4%) relaping. Repeat peritonitis episodes occurred 3.8 (1–12) months after the previous episode. Of the repeat episodes, 24 (68.6%) developed within 6 months after antibiotic was completed for the previous episode. The control group was 77 (69.3%) patients who never developed repeat and/or relapsing peritonitis. The clinical characteristics of the patients at the time of peritonitis are summarized in Table 1.

There is no significant difference in the baseline clinical characteristics between groups. Diabetic patients were marginally more common in the control group (23.3% versus 8.3%) but did not reach statistical significance.

### 3.2. Causative Microorganism

There was a significant difference in the distribution of the causative organisms between groups (Table 2). Gram-positive peritonitis is more common in the repeat/relapsing group especially *Streptococci viridans* (43.5% versus 21.3%, *p*=0.01). A higher percentage of peritonitis episodes was seen in the control group due to Gram-negative bacteria (26.6% vs. 9.0%, *p*=0.02).

### 3.3. Exit-Site Infection

Concomitant exit-site infection was present in 13.2% control episodes and in 5.9% of the repeat/relapsing group, but these results were not statistically significant (*p*=0.16).

### 3.4. Clinical Response

The major clinical outcomes are summarized in Table 3. Compared with the control group, the repeat/relapsing group had a significantly higher cure rate (80.4% *versus* 74.5%, *p*=0.01), lower rate of hospitalization (10.9% versus 27.7%, *p* = 0.01), and marginally lower permanent transfer to hemodialysis (10.9% *versus* 17.0%, *p*=0.18). The rate of catheter removal (26.1% *versus* 28.7%, *p*=0.74) was similar between the groups. In our program, there was only one death in the control group.

**Figure 2:**
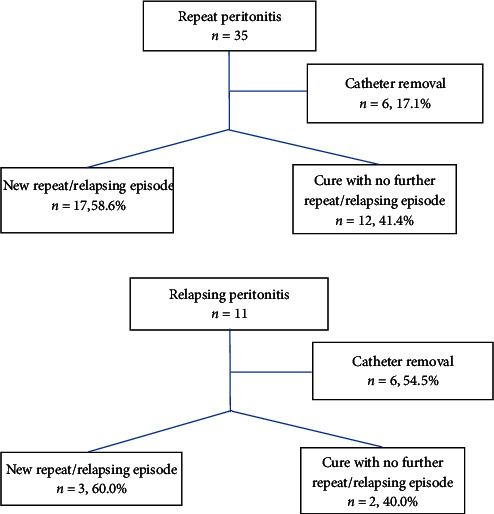
Repeat and relapsing peritonitis clinical outcomes with and without catheter removal.

A subanalysis of repeat and relapsing peritonitis clinical response was performed (Figure 2). On repeat peritonitis when PD catheter was not removed, 58.6% developed a new repeat/relapsing episode. This result is similar for relapsing episodes since 60.0% also developed a new repeat/relapsing episode when the PD catheter was not removed.

### 3.5. Technique Survival

**Figure 3:**
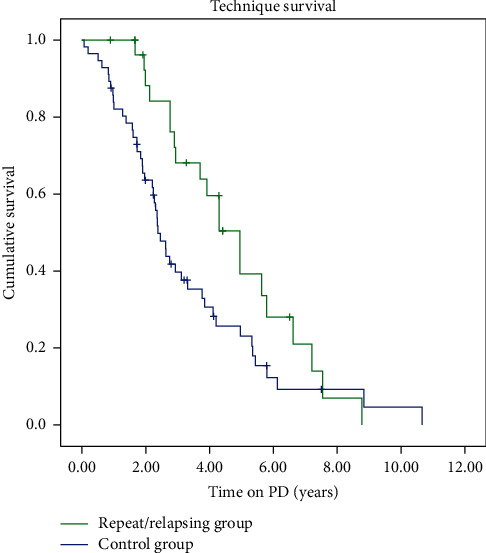
Technique survival repeat/relapsing *versus* control group.

Figure 3 shows technique survival for both groups. Technique survival for 1, 2, and 4 years were 100%; these were 84.1% and 59.6% for repeat/relapsing group and 82.0%, 61.6%, and 28.2% for the control groups (log rank = 4.5, *p*=0.03).

## 4. Discussion

In this retrospective study, baseline demographic and comorbidities were similar between groups, and it seems that the development of repeat/relapsing episodes is independent of host characteristics. We found that *Streptococcus viridans* was the predominant causative agent in the repeat/relapsing group; Gram-negatives were seen in the control group. These findings are different from previous reports that described *Staphylococcus aureus* and coagulase-negative *Staphylococcus* as the most prevalent microorganisms in repeat/relapsing peritonitis [4, 8]. Surveillance of S*taphylococcus aureus* carriage status in our program and treatment of carriers with mupirocin may explain this difference and is supported by a retrospective analysis of 104 *Streptococcus* peritonitis that showed a predominance of streptococcus in repeat/relapsing peritonitis after the use of mupirocin in daily exit-site care [9, 10]. Although there was a marginal predominance of exit-site infection in the repeat/relapsing group, it did not reach statistical significance difference, which is in line with other studies [4].

The clinical response is also different: the repeat/relapsing group also shows a higher cure rate and less hospitalization. One possible explanation is that Gram-negative and fungal peritonitis—all with less favorable therapeutic response and a higher probability of prompt catheter removal—did not occur in the repeat/relapsing group [11, 12].

The catheter removal rate was similar between the repeat/relapsing group and control group, but permanent transfer (10.9% versus 17.0%) and temporary transfer to hemodialysis after a peritonitis episode (10.6% versus 8.7%) were more frequent in control group though it did not reach statistical significance. One possible explanation is that control group had more severe peritonitis episodes by Gram-negative and fungi that led to prompt catheter removal and transfer to hemodialysis. The cure rate was higher in the repeat/relapsing group, and it was possible to remove and reinsert the PD catheter as a single procedure under antibiotic coverage and avoid hemodialysis transference.

When Tenckhoff catheter was not removed, the results were similar between repeat and relapsing peritonitis: in the repeat peritonitis group, 68.6% developed a subsequent relapse or repeat episode by the same microorganism *versus* 60.0% in the relapsing group. This result questions if a repeat peritonitis episode should be a formal indication to catheter removal as it is in relapsing ones.

Technique survival was better in the group with relapsing/repeat episodes than the control group. We emphasize that there were no significant differences between the groups in terms of age, PD modality, or comorbidities; thus, the type of peritonitis seems to have an impact on technique survival. Our practice since 2004 was to remove the PD catheter after the first repeat peritonitis episode. This could explain why repeat and relapsing peritonitis groups offer superior technique survival.

This study has some limitations: it is a single-center retrospective study with a small sample size. We also assumed that repeat and relapsing peritonitis could belong to the same group because the mechanism that causes subsequent episodes in both cases is believed to be the same. Over time, there were changes in antibiotic protocol, exit-site care, and timing of catheter removal that could introduce some heterogeneity to the results.

## 5. Conclusion

Although repeat and relapsing peritonitis have a higher cure rate, they lead to further episodes of peritonitis and consequent morbidity. When Tenckhoff catheter was not removed, the probability of another peritonitis episode by the same microorganism is similar in repeat and relapsing peritonitis.

## Figures and Tables

**Table 1:** Clinical characteristics of the repeat/relapsing and control group at the time of peritonitis.

	Repeat/relapsing group (*N* = 36)	Control group (*N* = 77)	*p* value
Age, media [IQR]	58, [47, 63.8]	55 [42, 64.5]	0.64
Male sex, *n*, %	21, 58.3%	46, 59.7%	0.89
CAPD, *n*, %	26, 72.2%	54, 70.1%	0.82

CKD cause			0.49
Glomerulonephritis, *n*%	14, 38.9%	32, 41.6%	
Diabetes, *n*%	3, 8.3%	18, 23.3%	
Polycystic kidney disease, *n*%	3, 8.3%	8, 10.4%	
Obstruction, *n*%	2, 5.6%	6, 7.8%	
Others/unknown, *n*%	14, 38.8%	13, 16.8%	

Major comorbidities			
Coronary heart disease, *n*%	4, 11.1%	13, 16.9%	0.42
Peripheral arterial disease, *n*%	1, 2.8%	8, 10.4%	0.16
Cerebrovascular disease, *n*%	5, 13.9%	5, 6.5%	0.20
Diabetes mellitus, *n*%	3, 8.3%	18, 23.3%	0.06

CAPD—continuous ambulatory peritoneal dialysis; CKD—chronic kidney disease; IQR—interquartile range.

**Table 2:** Causative microorganisms of the repeat/relapsing and control group.

	Repeat/relapsing peritonitis *N* = 46	Control peritonitis *N* = 94	*p* value
*Streptococci viridans*	20, 43.5%	20, 21.3%	0.01
Coagulase-negative *Staphylococcus*	14, 30.4%	21, 22.3%	0.38
*Staphylococcus aureus*	8, 17.4%	17, 18.1%	0.78
*Enterococcus* spp.	0, 0%	3, 3.2%	0.55
*Corynebacterium* spp.	0, 0%	3, 3.2%	0.55
Gram negative	4, 9.0%	25, 26.6%	0.02
*Pseudomonas* spp.	0	7, 7.4%	0.96
Fungi	0, 0%	5, 5.3%	0.32

**Table 3:** Repeat/relapsing and control group clinical response.

	Repeat/relapsing peritonitis *N* = 46	Control peritonitis *N* = 94	*p* value
Cure, *n*%	37, 80.4%	70, 74.5%	0.01
Catheter removal, *n*%	12, 26.1%	27, 28.7%	0.74
Hospitalization, *n*%	5, 10.9%	26, 27.7%	0.01
Temporary transfer to HD, *n*%	4, 8.7%	10, 10.6%	0.68
Permanent transfer to HD, *n*%	5, 10.9%	16, 17.0%	0.18
Death, *n*%	0, 0%	1, 1.1%	0.72

## Data Availability

The data that support the findings of this study are available from the corresponding author upon reasonable request.

## References

[1] Chaudhary K. (2011). Peritoneal dialysis drop-out: causes and prevention strategies. *International Journal of Nephrology*.

[2] Szeto C.-C., Li P. K.-T. (2019). Peritoneal dialysis-associated peritonitis. *Clinical Journal of the American Society of Nephrology*.

[3] Li P. K.-T., Szeto C. C., Piraino B. (2016). ISPD Peritonitis recommendations: 2016 update on prevention and treatment. *Peritoneal Dialysis International: Journal of the International Society for Peritoneal Dialysis*.

[4] Szeto C.-C., Ching-Ha Kwan B., Chow K.-M. (2011). Repeat peritonitis in peritoneal dialysis: retrospective review of 181 consecutive cases. *Clinical Journal of the American Society of Nephrology*.

[5] Finkelstein E. S., Jekel J., Troidle L., Gorban-Brennan N., Finkelstein F. O., Bia F. J. (2002). Patterns of infection in patients maintained on long-term peritoneal dialysis therapy with multiple episodes of peritonitis. *American Journal of Kidney Diseases*.

[6] Nessim S. J., Nisenbaum R., Bargman J. M., Jassal S. V. (2012). Microbiology of peritonitis in peritoneal dialysis patients with multiple episodes. *Peritoneal Dialysis International: Journal of the International Society for Peritoneal Dialysis*.

[7] Read R. R., Eberwein P., Dasgupta M. K. (1989). Peritonitis in peritoneal dialysis: bacterial colonization by biofilm spread along the catheter surface. *Kidney International*.

[8] Szeto C.-C., Kwan B. C.-H., Chow K.-M. (2008). Coagulase negative staphylococcal peritonitis in peritoneal dialysis patients: review of 232 consecutive cases. *Clinical Journal of the American Society of Nephrology*.

[9] Piraino B., Bernardini J., Brown E. (2011). ISPD position statement on reducing the risks of peritoneal dialysis-related infections. *Peritoneal Dialysis International: Journal of the International Society for Peritoneal Dialysis*.

[10] Shukla A., Abreu Z., Bargman J. M. (2006). Streptococcal PD peritonitis—a 10-year review of one centre’s experience. *Nephrology Dialysis Transplantation*.

[11] Szeto C.-C., Chow K.-M. (2007). Gram-negative peritonitis-the achilles heel of peritoneal dialysis?. *Peritoneal Dialysis International: Journal of the International Society for Peritoneal Dialysis*.

[12] Auricchio S., Giovenzana M. E., Pozzi M. (2018). Fungal peritonitis in peritoneal dialysis: a 34-year single centre evaluation. *Clinical Kidney Journal*.

